# Ropeginterferon dose-escalation strategies in polycythemia vera: a systematic review and meta-analysis

**DOI:** 10.1186/s12885-026-15939-x

**Published:** 2026-04-11

**Authors:** Seug Yun Yoon, Suyeon Park, Sun Young Jeong, Min-Young Lee, Kyoung Ha Kim, Namsu Lee, Jong-Ho Won, Sung-Eun Lee

**Affiliations:** 1 Division of Hematology & Medical Oncology, Department of Internal Medicine, Soonchunhyang University Seoul Hospital, Seoul, Republic of Korea; 2 Department of Biostatistics, Soonchunhyang University Seoul Hospital, Seoul, Republic of Korea; 3 Department of Applied Statistics, Chung-Ang University, Seoul, Republic of Korea; 4 Department of Hematology, Seoul St. Mary’s Hospital, College of Medicine, The Catholic University of Korea, 222 Banpodae-ro, Seocho-Gu, Seoul, 06591 Republic of Korea

**Keywords:** Ropeginterferon alfa-2b, Polycythemia vera, Dose-escalation, Hematologic response, Molecular response

## Abstract

**Background:**

Ropeginterferon alfa-2b is increasingly used as a long-acting interferon therapy for polycythemia vera (PV), providing hematologic and molecular benefits. However, clinical studies have implemented different dose-escalation strategies, and their impact on outcomes has not been systematically evaluated.

**Methods:**

A systematic search of PubMed, Embase, and the Cochrane Library (September 24, 2025) identified studies reporting clinical outcomes of ropeginterferon in PV. Nine studies met eligibility criteria. Two reviewers independently extracted data, and meta-analyses were conducted using random-effects models. Subgroup analyses compared slow dose-up (SDU) and rapid dose-up (RDU) regimens at 1-, 2-, and 3-year follow-up when available.

**Results:**

The pooled 1-year complete hematologic response (CHR) rate was 0.59 (95% CI, 0.47–0.71). RDU regimens yielded significantly higher CHR than SDU at both 1 year (0.67 vs. 0.41; *p* < 0.001) and 2 years (0.75 vs. 0.63; *p* = 0.038). Molecular response (MR) also favored RDU at 1 year (0.59 vs. 0.36; *p* < 0.001), with differences diminishing at 2 years. The pooled 1-year reduction in JAK2 V617F allele burden was − 22.2% (95% CI, − 34.2% to − 10.2%; *p* < 0.001). Safety outcomes were favorable, with low rates of thrombosis (4%), serious adverse events (5%), and treatment discontinuation (7%).

**Conclusions:**

Ropeginterferon alfa-2b provides meaningful hematologic and molecular responses with an acceptable safety profile in PV. Rapid dose-escalation facilitates earlier CHR and MR without increasing toxicity, suggesting titration speed as an important determinant of early treatment optimization.

**Supplementary Information:**

The online version contains supplementary material available at 10.1186/s12885-026-15939-x.

## Introduction

Polycythemia vera (PV) is a chronic myeloproliferative neoplasm driven primarily by the *JAK2* V617F mutation [1]. The main therapeutic goal in PV is to prevent thrombotic complications through adequate cytoreduction and hematocrit control. Although hydroxyurea has historically been considered a first-line cytoreductive therapy, evolving treatment recommendations and emerging clinical data have increasingly supported interferon-based strategies, particularly in younger patients and in those for whom disease modification is a therapeutic objective. Interferon (IFN) has long served as an alternative with potential disease-modifying activity [2]. However, conventional IFN formulations require frequent injections and are often limited by treatment-related adverse effects, resulting in poor tolerability in clinical practice. The development of ropeginterferon alfa-2b, a novel monopegylated IFN with an extended half-life, has renewed interest in IFN therapy for PV. Its less frequent dosing schedule and favorable safety profile have made it an attractive long-term treatment option. Importantly, ropeginterferon has been shown to reduce *JAK2* V617F allele burden, suggesting possible disease modification beyond hematologic control [3]. Sustained reductions in mutant allele burden may reflect suppression of the malignant clone and suggest the potential for long-term disease control beyond cytoreduction.

Although several systematic reviews have evaluated interferon-based therapies in PV, none have examined whether different dose-escalation strategies influence clinical outcomes with ropeginterferon alfa-2b. Therefore, we conducted a systematic review and meta-analysis to provide an updated synthesis of clinical outcomes associated with ropeginterferon therapy in PV and to assess the potential impact of dose-escalation strategies on efficacy and safety where data permitted.

## Methods

### Systematic review

**Fig. 1:**
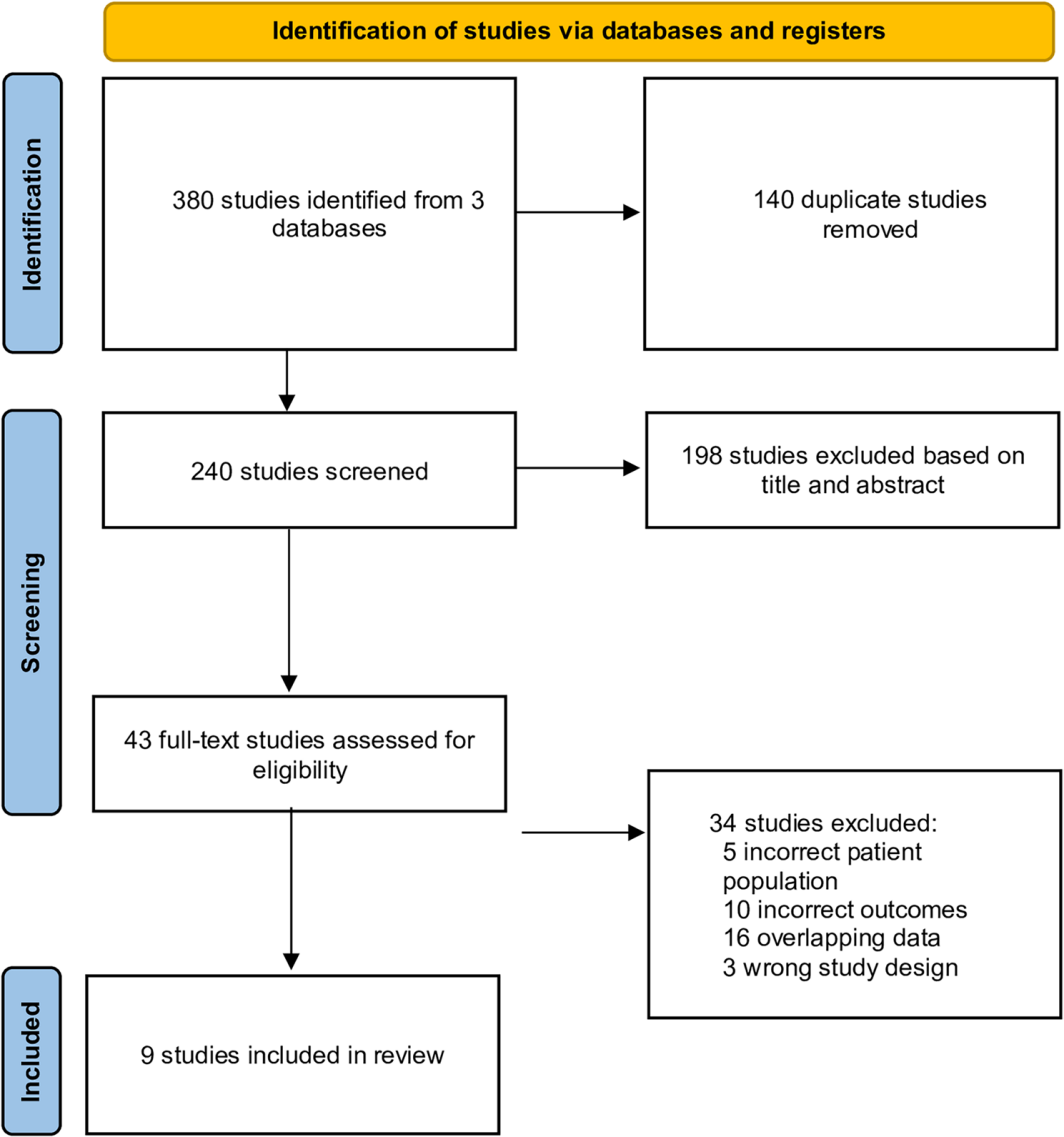
PRISMA flowchart of study selection process. PRISMA; Preferred Reporting Items for Systematic Reviews and Meta-Analyses. *Consider, if feasible to do so, reporting the number of records identified from each database or register searched (rather than the total number across all databases/registers). **If automation tools were used, indicate how many records were excluded by a human and how many were excluded by automation tools

A systematic literature search was conducted in PubMed, Embase, and the Cochrane Library on September 24, 2025, using predefined terms related to polycythemia vera and ropeginterferon alfa-2b. Reference lists of eligible articles and prior reviews were also screened to ensure completeness. The full electronic search strategies for all databases are provided in the Supplementary Materials (Table S1 – S3). A total of 380 records were identified. After removal of 140 duplicates, 240 records underwent title and abstract screening. Among these, 43 full-text articles were assessed for eligibility, and 9 studies met the final inclusion criteria (Table 1). The study selection process is summarized in Fig. 1. All stages of screening—including title/abstract review and full-text assessment—were performed independently by two reviewers (SY Yoon and SE Lee), and any discrepancies were resolved through discussion or adjudication by a third reviewer. Studies were excluded if they enrolled fewer than 10 PV patients, lacked extractable outcomes, used inappropriate study designs, or represented overlapping datasets. The risk of bias was assessed according to study design, with randomized trials evaluated using the RoB 2 tool and non-randomized studies assessed using the ROBINS-I tool. Detailed risk-of-bias findings are presented in Supplementary Table S4.

**Table 1:** Baseline characteristics of included studies

Study (Year)	Design / Country	*N* (PV)*	Median age	Male (%)	JAK2 V617F (%)	Hct (%)	WBC (×10⁹/L)	Plt (×10⁹/L)	Prior HU	Dose schedule	Starting dose	Escalation	Max dose
Gisslinger et al. (2020) [4]	RCT / Europe	127	60.0	46%	41.9%	47.1	10.6	485	35%	SDU	50–100 µg	+ 50 µg q2wk	540 µg
Barbui et al. (2021) [5]	RCT / Italy	50	51.4	74%	30.0%	44.2	10.8	645	0%	Fix dose	100 µg		100 µg
Yoon et al. (2025) [6]	Phase 2 / Korea	95	58	54%	69.9%	49.6	13.2	545	45%	RDU	250 µg	250→350→500	500 µg
Palandri et al. (2024) [7]	Retrospective / Italy	18	55	72%	43.7%	NA	7.5	490	78%	SDU-like	100 µg	variable	200 µg
SUO et al. (2024) [8]	Phase 2 / China	49	53.0	63%	58.5%	46.0	11.4	478.5	100%	RDU	250 µg	250→350→500	500 µg
Chang et al. (2025) [9]	Retrospective / Taiwan	15	63.8	40%	NA	47	20.1	627	60%	Mixed	50–250 µg	variable	500 µg
Edahiro et al. (2022) [10]	Phase 2 / Japan	29	54	44.8%	72%	NA	NA	NA	51.7%	SDU	50 µg	+ 50 µg q2wk	500 µg
Chen et al. (2024) [11]	Retrospective / Taiwan	15	49.8	53.3%	71.1%	NA	NA	NA	26.7%	RDU	250 µg	250→350→500	500 µg
Gisslinger et al. (2015) [12]	Phase 1/2 / Austria	51	56	61%	41%	44.8	11.1	429	33%	SDU	50 µg	+ 50 µg q2wk	540 µg

*PV* Polycythemia vera, *RCT* Randomized controlled trial, *HU* Hydroxyurea, *SDU* Slow dose-up, *RDU* Rapid dose-up, *WBC* White blood cell count, *Plt* Platelet count, *Hct* Hematocrit, *VAF* Variant allele fraction, *NA* Not available

*Data were restricted to individuals diagnosed with polycythemia vera who received ropeginterferon alfa-2b as part of their treatment regimen

### Eligibility criteria

Studies were included if they met all of the following criteria:

1. Adult patients (≥ 18 years) diagnosed with polycythemia vera.
2. Treatment with ropeginterferon alfa-2b.
3. Reported outcomes including CHR, MR, JAK2 V617F allele burden, adverse events, treatment discontinuation, or disease transformation.
4. Study design involving randomized trials, non-randomized comparative studies, prospective or retrospective cohorts, or single-arm interventional studies.

Case reports, reviews, pharmacokinetic-only studies, and studies without sufficient data were excluded.

### Dose-escalation strategies

Studies were categorized into slow dose-up (SDU) and rapid dose-up (RDU) groups.

- SDU regimens started at 50–100 µg, with 50 µg increases every two weeks up to 500 µg.
- RDU regimens began at 250 µg and were escalated stepwise (250 → 350 → 500 µg) at two-week intervals, followed by maintenance at 500 µg every two weeks.

Dose-escalation classifications were based on dosing schedules explicitly reported in each study.

### Data extraction

Two independent reviewers extracted study characteristics, dosing regimens, follow-up duration, definitions of CHR and MR, *JAK2* V617F allele burden, adverse events, discontinuation, and transformation outcomes using a standardized form. Any disagreements were resolved by consensus or by involving a third reviewer. Outcome definitions were extracted as reported in each study and aligned with ELN criteria where applicable. When definitions differed across studies, outcomes were harmonized to the most commonly applied criteria when appropriate.

### Meta-analysis

All meta-analyses were conducted using R (version 4.5.1) with the ‘metafor’ package. Summary statistics for binary outcomes were expressed as proportions (PR) and analyzed using a random-effects model, with between-study variance estimated via the restricted maximum likelihood (REML) method. Continuous outcomes were analyzed as mean differences (MD) under a random-effects model. For safety outcomes with zero events, a continuity correction of 0.5 was applied, and the proportion logit (PLO) transformation was used to stabilize variances in sparse data.

Subgroup analyses were conducted based on dose-escalation strategy, categorizing the included studies into the SDU and RDU groups. Follow-up analyses were performed at 1-, 2-, and 3-year intervals to assess temporal trends in outcomes. Long-term outcomes were further assessed by extracting 2- and 3-year CHR and MR data from studies that provided extended follow-up under either SDU or RDU dosing strategies. These extended follow-up data were not used as eligibility criteria but were incorporated into subgroup analyses when available. A random-effects model was applied to test for subgroup differences and to determine whether pooled estimates differed significantly between the two dosing groups at time points for which data were available for both groups. Because only the SDU group contributed data at the 3-year follow-up, no between-group comparison was conducted for that interval. Publication bias was evaluated using Begg’s rank correlation test. All analyses were two-sided, with p-values < 0.05 considered statistically significant.

## Results

A total of nine studies met the eligibility criteria and were included in the final analysis (Fig. 1). The overall risk-of-bias assessment is summarized in Supplementary Table S4. Among the randomized controlled trials (RCTs, *n* = 2), both were rated as low risk with respect to the randomization process and outcome measurement. One study showed some concerns regarding deviations from intended interventions, missing outcome data, and selection of reported results, whereas the other demonstrated low risk across these domains.

For non-randomized studies of interventions (NRSIs, *n* = 7), all were judged to have a serious overall risk of bias, primarily due to confounding and participant selection. Other domains—including classification of interventions, deviations from intended interventions, missing data, outcome assessment, and reporting selection—were generally rated as low to moderate risk. Overall, RCTs exhibited lower risk of bias compared with NRSIs, which were more susceptible to confounding and selection-related bias.

### Complete Hematologic Response (CHR)

**Fig. 2:**
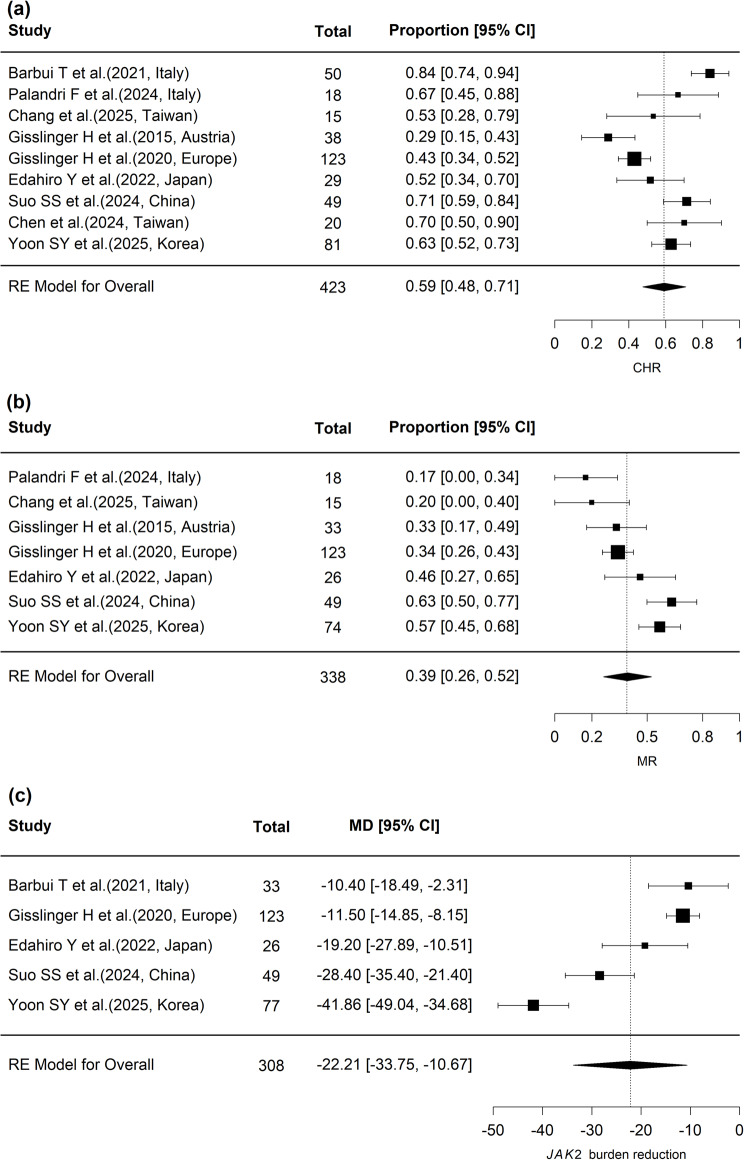
Pooled estimates of clinical outcomes with ropeginterferon alfa-2b in polycythemia vera. **A** Forest plot of 1-year complete hematologic response (CHR). Individual study estimates and 95% confidence intervals (CIs) are shown, with the pooled proportion calculated using a random-effects model. **B** Forest plot of 1-year molecular response (MR). Proportions with 95% CIs are displayed for each study, along with the pooled random-effects estimate. **C** Forest plot of absolute reduction in JAK2 V617F allele burden at 1 year. Mean differences (MDs) with 95% CIs are shown for each study, and a pooled MD is presented using a random-effects model. * CHR, complete hematologic response; MR, molecular response; MD, mean difference

**Fig. 3:**
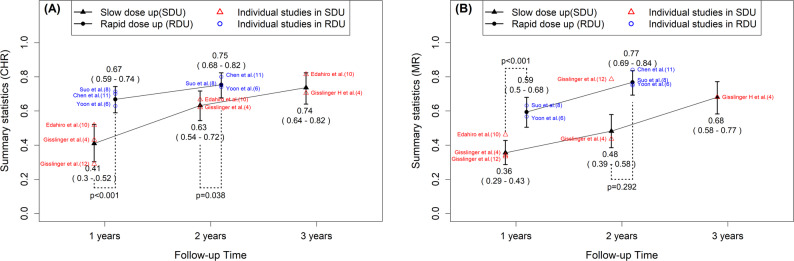
Subgroup meta-analysis of dose-escalation strategies on clinical outcomes. **A** Complete hematologic response (CHR) and (**B**) molecular response (MR) at 1-, 2-, and 3-year follow-up comparing slow dose-up (SDU) and rapid dose-up (RDU) regimens. * SDU, slow dose-up; RDU, rapid dose-up; CHR, complete hematologic response; MR, molecular response

At the 1-year follow-up, nine studies were included, with an overall complete hematologic response (CHR) proportion of 0.59 (95% CI, 0.47–0.71; *p* < 0.001) and substantial heterogeneity (I² = 83.0%, *p* < 0.001) (Fig. 2a). No evidence of publication bias was detected, as indicated by Begg’s rank test (*p* = 1.000). In subgroup analyses, the CHR proportion was 0.41 (3 studies; 95% CI, 0.30–0.52; *p* < 0.001) for the slow dose-up (SDU) group and 0.67 (3 studies; 95% CI, 0.59–0.74; *p* < 0.001) for the rapid dose-up (RDU) group, and the difference between the two groups was statistically significant at the 1-year follow-up (*p* < 0.001) (Fig. 3a). At the 2-year follow-up, subgroup CHR proportions were 0.63 (2 studies; 95% CI, 0.54–0.72; *p* < 0.001) for SDU and 0.75 (3 studies; 95% CI, 0.68–0.82; *p* < 0.001) for RDU, with a statistically significant difference between the groups (*p* = 0.038) (Fig. 3a). At the 3-year follow-up, data were available only for the SDU group (2 studies; CHR 0.74, 95% CI, 0.64–0.82; *p* < 0.001), and no between-group comparison was performed due to the limited availability of data.

### Molecular Response (MR)

At the 1-year follow-up, seven studies were included, yielding a pooled MR proportion of 0.40 (95% CI, 0.28–0.53; *p* < 0.001) with substantial heterogeneity (I² = 78.3%, *p* < 0.001) (Fig. 2b). No evidence of publication bias was observed (Begg’s rank test, *p* = 0.453). In subgroup analyses, the MR proportion was 0.36 (3 studies; 95% CI, 0.29–0.43; *p* < 0.001) for the SDU group and 0.59 (2 studies; 95% CI, 0.50–0.68; *p* < 0.001) for the RDU group, with a statistically significant difference between the groups (*p* < 0.001) (Fig. 3b). At the 2-year follow-up, subgroup MR proportions were 0.48 (2 studies; 95% CI, 0.39–0.58; *p* < 0.001) for SDU and 0.77 (3 studies; 95% CI, 0.69–0.84; *p* < 0.001) for RDU, with no statistically significant difference between the groups at this time point (*p* = 0.292) (Fig. 3b). At the 3-year follow-up, only one study (Gisslinger H et al., 2020, Europe) reported data, showing an MR proportion of 0.68 (95% CI, 0.58–0.77; *p* < 0.001); thus, no subgroup comparison was performed due to the limited data.

### *JAK2* allele burden reduction

A meta-analysis including five studies evaluated the absolute change in *JAK2* allele burden at 1 year. The pooled estimate showed a significant reduction of − 22.20% (95% CI, − 34.19% to − 10.21%; *p* < 0.001). Substantial heterogeneity was detected among studies (I² = 95.2%, Q = 83.15, *p* < 0.001) (Fig. 2c). Assessment of publication bias using Begg’s rank correlation test revealed no significant bias (*p* = 0.624). In subgroup analyses, the mean reduction was − 14.26% in the SDU group and − 35.11% in the RDU group, with a statistically significant difference between subgroups (*p* = 0.0066; Supplementary Table S5).

### Safety outcomes

**Fig. 4:**
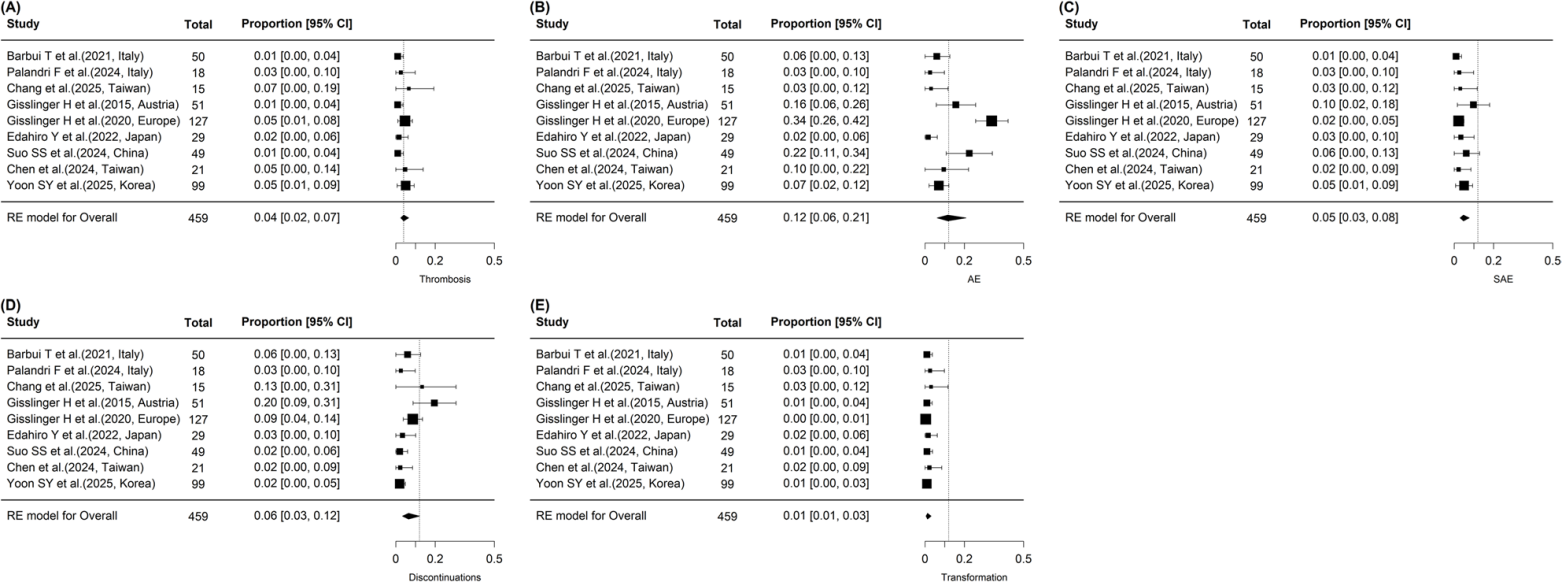
Safety outcomes associated with ropeginterferon alfa-2b therapy in polycythemia vera. **A** Forest plot of thrombosis incidence. Individual study estimates and 95% confidence intervals (CIs) are shown, with pooled proportions calculated using a random-effects model. **B** Forest plot of overall adverse events (AEs), including both hematologic and non-hematologic events as reported in each study. **C** Forest plot of serious adverse events (SAEs). Pooled incidence estimates with 95% CIs were generated using random-effects modeling. **D** Forest plot of treatment discontinuations attributed to adverse events or intolerance. **E** Forest plot of progression to advanced myeloproliferative neoplasms, including post-PV myelofibrosis or acute myeloid leukemia. * All summary estimates were obtained using random-effects models with logit-transformed proportions (PLO) to stabilize variance. ** CI, confidence interval; AE, adverse event; SAE, serious adverse event

A total of nine studies were included in the safety analysis (Fig. 4). Adverse events (AEs) included both hematologic and non-hematologic events as reported in each study. The pooled incidence of thrombosis was 4.0% (95% CI, 2.0–7.0%; *p* < 0.001), with no significant heterogeneity observed (I² = 0.0%, Q = 4.315, *p* = 0.828). The overall incidence of AEs was 11.0% (95% CI, 6.0–21.0%; *p* < 0.001), with substantial heterogeneity (I² = 79.5%, Q = 39.028, *p* < 0.001). Serious adverse events (SAE) occurred in 5.0% overall (95% CI, 3.0–8.0%; *p* < 0.001), with no significant heterogeneity (I² = 0.0%, Q = 6.442, *p* = 0.597). Treatment discontinuations occurred in 7.0% overall (95% CI, 4.0–12.0%; *p* < 0.001), showing moderate heterogeneity (I² = 54.2%, Q = 17.475, *p* = 0.026). Transformation to advanced disease was rare, with an overall incidence of 1.0% (95% CI, 1.0–3.0%; *p* < 0.001), and no heterogeneity observed (I² = 0.0%, Q = 1.744, *p* = 0.988). In subgroup analyses according to dose-escalation strategy, discontinuation rates were 11% in the SDU group and 2% in the RDU group, without a statistically significant difference between subgroups. Similarly, the incidences of thrombosis, serious adverse events, and transformation were comparable between SDU and RDU groups (Supplementary Table S5). Begg’s rank correlation test indicated no significant publication bias for all outcomes, with p-values ranging from 0.211 to 0.532, except for transformation, which demonstrated significant publication bias (*p* < 0.001).

## Discussion

In this systematic review and meta-analysis of nine studies, ropeginterferon alfa-2b demonstrated meaningful hematologic and molecular responses with a favorable safety profile in patients with PV. A key finding of this analysis was that dose-escalation strategy appears to influence the timing and depth of treatment response. RDU regimens produced substantially higher 1-year CHR and MR rates compared with SDU strategies, and this advantage persisted into the 2-year follow-up. These observations suggest that titration speed may play an important role in optimizing the early therapeutic effects of ropeginterferon.

These findings may have important clinical implications. Earlier achievement of CHR could potentially contribute to earlier reduction in thrombotic risk, as delayed hematocrit control has been associated with increased thrombosis in PV [13]. Similarly, more rapid molecular responses may increase the likelihood of long-term disease modification, given prior observations linking deeper reductions in *JAK2* V617F allele burden with more favorable disease trajectories [14]. Importantly, the accelerated titration used in RDU regimens did not appear to increase treatment-limiting toxicity, suggesting that faster dose-escalation may be feasible without compromising tolerability. Reflecting these emerging data, recent randomized controlled trials have increasingly adopted RDU regimens, and clinical studies directly comparing SDU and RDU strategies are now underway [15, 16].

Previous systematic reviews summarized the overall efficacy and safety of ropeginterferon but did not investigate whether dosing strategies influence clinical outcomes [17, 18]. By integrating data across different titration approaches and evaluating outcomes at multiple time intervals, our study provides the first systematic evidence suggesting that faster dose-escalation may enhance early hematologic and molecular responses without compromising safety. During data harmonization, we also observed inconsistencies in outcome definitions and reported population sizes across studies and prior reviews. Although these discrepancies should be interpreted with caution, they highlight the need for standardized reporting in future trials.

Safety outcomes in this study were generally favorable. The pooled incidence of thrombosis was low, and SAEs were infrequent, occurring in approximately 5% of patients. Overall adverse events and discontinuation rates were also modest, and no clear differences were observed between SDU and RDU strategies in the available data. Although the studies included were heterogeneous in design and reporting, the overall safety profile aligns with previous clinical experience, supporting the tolerability of ropeginterferon across a range of dosing strategies.

The clinical context of interferon therapy in PV has also evolved. Conventional interferon formulations are now rarely used in practice, and pegylated interferon products—once widely utilized in hepatitis treatment—have experienced global supply limitations following the introduction of direct-acting antiviral agents [19]. Consequently, access to peginterferon has become restricted in many regions. In this setting, ropeginterferon alfa-2b represents the most widely available and practical interferon-based option for PV, supported by its long-acting formulation, consistent availability, and accumulating evidence of both hematologic and molecular efficacy. These factors highlight the increasingly central role of ropeginterferon in the modern management of PV [3].

This study has several limitations. First, most included studies were single-arm cohorts, and only a limited number of randomized trials were available, which may restrict the strength of comparative inferences. Second, variability in outcome definitions and reporting across studies required harmonization and may contribute to residual heterogeneity. Third, the geographic distribution of dosing strategies differed across studies, with RDU regimens predominantly reported from Asian cohorts whereas SDU regimens were largely based on European or Japanese cohorts, raising the possibility of region-specific practice patterns influencing outcomes. Fourth, although we evaluated SDU versus RDU regimens, the number of studies providing long-term outcomes—particularly at 3 years—was limited, reducing the robustness of comparisons at extended follow-up. Additionally, time-to-molecular-response outcomes were not consistently reported across studies, precluding formal comparisons of median response time between SDU and RDU strategies. Finally, publication bias could not be excluded for some outcomes despite formal testing.

In conclusion, this systematic review and meta-analysis demonstrates that ropeginterferon alfa-2b is an effective and well-tolerated therapy for PV. Importantly, rapid dose-escalation strategies appear to facilitate earlier achievement of hematologic and molecular responses without compromising safety, suggesting that titration speed may be an important determinant of early treatment optimization. As ongoing randomized trials continue to clarify the comparative effects of different dosing strategies, these findings provide a foundation for refining ropeginterferon dosing approaches in clinical practice and support its expanding role in the long-term management of PV.

## Supplementary Information

Supplementary Material 1.

## Data Availability

All data generated or analyzed during this study are included in this published article and its supplementary information files.

## Abbreviations

PV: Polycythemia vera
IFN: Interferon
SDU: Slow dose-up
RDU: Rapid dose-up
CHR: Complete hematologic response
MR: Molecular response
MD: Mean difference
RCT: Randomized controlled trial
NRSI: Non-randomized studies of intervention
CI: Confidence interval
AE: Adverse event
SAE: Serious adverse event
WBC: White blood cell count
Plt: Platelet count
Hct: Hematocrit
VAF: Variant allele fraction
NA: Not available
