# Supplementary material for: Ropeginterferon dose-escalation strategies in polycythemia vera: a systematic review and meta-analysis
Source: BMC Cancer. 2026 Apr 11;26:641. doi: 10.1186/s12885-026-15939-x (PMC13188781; doi:10.1186/s12885-026-15939-x)
Supplement: Supplementary file 1 — Supplementary Material 1. [file 12885_2026_15939_MOESM1_ESM.pdf]

Supplementary Materials

Table of Contents

Supplementary Table S1. PubMed search strategy

Supplementary Table S2. Embase search strategy

Supplementary Table S3. Cochrane Library search strategy

Supplementary Table S4. Risk-of-bias assessment of included studies (RoB 2 and ROBINS-I)

Supplementary Table S5. Subgroup analyses comparing slow dose-up (SDU) and rapid dose-up (RDU) strategies for efficacy and safety outcomes

Supplementary Table S6. PRISMA Checklist

Table S1. Search strategy for PubMed (Search date: September 24, 2025)

Search number	Query	Search Details	Results
1	Polycythemia Vera[MeSH Terms]	"polycythemia vera"[MeSH Terms]	6,814
2	Polycythemia[MeSH Terms]	"polycythemia"[MeSH Terms]	6,280
3	"polycythaemia vera*"[Title/Abstract]	"polycythaemia vera*"[Title/Abstract]	1,070
4	"polycythemia vera*"[Title/Abstract]	"polycythemia vera*"[Title/Abstract]	6,740
5	"vaquez disease*"[Title/Abstract]	"vaquez disease*"[Title/Abstract]	95
6	erythraemia*[Title/Abstract]	"erythraemia*"[Title/Abstract]	38
7	erythremia*[Title/Abstract]	"erythremia*"[Title/Abstract]	270
8	polycythaemia*[Title/Abstract]	"polycythaemia*"[Title/Abstract]	2,111
9	polycythemia*[Title/Abstract]	"polycythemia*"[Title/Abstract]	11,010
10	PV[Title/Abstract]	"PV"[Title/Abstract]	37,628
11	#1 OR #2 OR #3 OR #4 OR #5 OR #6 OR #7 OR #8 OR #9 OR #10	"polycythemia vera"[MeSH Terms] OR "polycythemia"[MeSH Terms] OR "polycythaemia vera*"[Title/Abstract] OR "polycythemia vera*"[Title/Abstract] OR "vaquez disease*"[Title/Abstract] OR "erythraemia*"[Title/Abstract] OR "erythremia*"[Title/Abstract] OR "polycythaemia*"[Title/Abstract] OR "polycythemia*"[Title/Abstract] OR "PV"[Title/Abstract]	52,029
12	"ropeginterferon alfa 2b"[Title/Abstract]	"ropeginterferon alfa 2b"[Title/Abstract]	92
13	ropeg*[Title/Abstract]	"ropeg*"[Title/Abstract]	129
14	#12 OR #13	"ropeginterferon alfa 2b"[Title/Abstract] OR "ropeg*"[Title/Abstract]	129
15	#11 AND #14	("polycythemia vera"[MeSH Terms] OR "polycythemia"[MeSH Terms] OR "polycythaemia vera*"[Title/Abstract] OR "polycythemia vera*"[Title/Abstract] OR "vaquez disease*"[Title/Abstract] OR "erythraemia*"[Title/Abstract] OR "erythremia*"[Title/Abstract] OR "polycythaemia*"[Title/Abstract] OR "polycythemia*"[Title/Abstract] OR "PV"[Title/Abstract]) AND ("ropeginterferon alfa 2b"[Title/Abstract] OR "ropeg*"[Title/Abstract])	99
16	Animals[MeSH Terms]	"animals"[MeSH Terms]	28,341,250
17	Humans[MeSH Terms]	"humans"[MeSH Terms]	22,964,080

18	#16 NOT #17	"animals"[MeSH Terms] NOT "humans"[MeSH Terms]	5,377,170
19	#15 NOT #18	(("polycythemia vera"[MeSH Terms] OR "polycythemia"[MeSH Terms] OR "polycythaemia vera*"[Title/Abstract] OR "polycythemia vera*"[Title/Abstract] OR "vaquez disease*"[Title/Abstract] OR "erythraemia*"[Title/Abstract] OR "erythremia*"[Title/Abstract] OR "polycythaemia*"[Title/Abstract] OR "polycythemia*"[Title/Abstract] OR "PV"[Title/Abstract]) AND ("ropeginterferon alfa 2b"[Title/Abstract] OR "ropeg*"[Title/Abstract])) NOT ("animals"[MeSH Terms] NOT "humans"[MeSH Terms])	99
20	#15 NOT #18	((("polycythemia vera"[MeSH Terms] OR "polycythemia"[MeSH Terms] OR "polycythaemia vera*"[Title/Abstract] OR "polycythemia vera*"[Title/Abstract] OR "vaquez disease*"[Title/Abstract] OR "erythraemia*"[Title/Abstract] OR "erythremia*"[Title/Abstract] OR "polycythaemia*"[Title/Abstract] OR "polycythemia*"[Title/Abstract] OR "PV"[Title/Abstract]) AND ("ropeginterferon alfa 2b"[Title/Abstract] OR "ropeg*"[Title/Abstract])) NOT ("animals"[MeSH Terms] NOT "humans"[MeSH Terms])) AND (english[Filter])	90

Filter applied: Humans; English language.

Table S2. Search strategy for Embase (Search date: September 24, 2025)

No.	Query	Search Details	Results
1	'polycythemia vera'/exp	'polycythemia vera'/exp	15,586
2	'polycythemia'/exp	'polycythemia'/exp	10,079
3	'polycythaemia vera*':ab,ti	'polycythaemia vera*':ab,ti	1,448
4	'polycythemia vera*':ab,ti	'polycythemia vera*':ab,ti	10,432
5	'vaquez disease*':ab,ti	'vaquez disease*':ab,ti	90
6	erythraemia*:ab,ti	erythraemia*:ab,ti	18
7	erythremia*:ab,ti	erythremia*:ab,ti	264
8	polycythaemia*:ab,ti	polycythaemia*:ab,ti	2,736
9	polycythemia*:ab,ti	polycythemia*:ab,ti	15,970
10	pv:ab,ti	pv:ab,ti	54,874
11	#1 OR #2 OR #3 OR #4 OR #5 OR #6 OR #7 OR #8 OR #9 OR #10	'polycythemia vera'/exp OR 'polycythemia'/exp OR 'polycythaemia vera*':ab,ti OR 'polycythemia vera*':ab,ti OR 'vaquez disease*':ab,ti OR erythraemia*:ab,ti OR erythremia*:ab,ti OR polycythaemia*:ab,ti OR polycythemia*:ab,ti OR pv:ab,ti	76,626
12	'ropeginterferon alpha2b'/exp	'ropeginterferon alpha2b'/exp	368
13	'ropeginterferon alfa 2b':ab,ti	'ropeginterferon alfa 2b':ab,ti	183
14	ropeg*:ab,ti	ropeg*:ab,ti	245
15	#12 OR #13 OR #14	'ropeginterferon alpha2b'/exp OR 'ropeginterferon alfa 2b':ab,ti OR ropeg*:ab,ti	406
16	#11 AND #15	('polycythemia vera'/exp OR 'polycythemia'/exp OR 'polycythaemia vera*':ab,ti OR 'polycythemia vera*':ab,ti OR 'vaquez disease*':ab,ti OR erythraemia*:ab,ti OR erythremia*:ab,ti OR polycythaemia*:ab,ti OR polycythemia*:ab,ti OR pv:ab,ti) AND ('ropeginterferon alpha2b'/exp OR 'ropeginterferon alfa 2b':ab,ti OR ropeg*:ab,ti)	247
17	'animal'/exp	'animal'/exp	36,600,851
18	'human'/exp	'human'/exp	30,230,991
19	#17 NOT #18	'animal'/exp NOT 'human'/exp	6,369,860
20	#16 NOT #19	(('polycythemia vera'/exp OR 'polycythemia'/exp OR 'polycythaemia vera*':ab,ti OR 'polycythemia vera*':ab,ti OR 'vaquez disease*':ab,ti OR erythraemia*:ab,ti OR erythremia*:ab,ti OR polycythaemia*:ab,ti OR polycythemia*:ab,ti OR pv:ab,ti) AND	246

		('ropeginterferon alpha2b'/exp OR 'ropeginterferon alfa 2b':ab,ti OR ropeg*:ab,ti)) NOT ('animal'/exp NOT 'human'/exp)	
21	#16 NOT #19 AND [english]/lim	(('polycythemia vera'/exp OR 'polycythemia'/exp OR 'polycythaemia vera*':ab,ti OR 'polycythemia vera*':ab,ti OR 'vaquez disease*':ab,ti OR erythraemia*:ab,ti OR erythremia*:ab,ti OR polycythaemia*:ab,ti OR polycythemia*:ab,ti OR pv:ab,ti) AND ('ropeginterferon alpha2b'/exp OR 'ropeginterferon alfa 2b':ab,ti OR ropeg*:ab,ti)) NOT ('animal'/exp NOT 'human'/exp) AND [english]/lim	240

Limit: humans; English language.

Table S3. Search strategy for the Cochrane Library (Search date: September 24, 2025)

ID	Search	Search Details	Hits
1	MeSH descriptor: [Polycythemia Vera] explode all trees	[mh "Polycythemia Vera"]	160
2	MeSH descriptor: [Polycythemia] explode all trees	[mh "Polycythemia"]	150
3	(polycythaemia next vera*):ab,ti	(polycythaemia next vera*):ab,ti	478
4	(polycythemia next vera*):ab,ti	(polycythemia next vera*):ab,ti	478
5	(vaquez next disease*):ab,ti	(vaquez next disease*):ab,ti	0
6	erythraemia*:ab,ti	erythraemia*:ab,ti	0
7	erythremia*:ab,ti	erythremia*:ab,ti	0
8	polycythaemia*:ab,ti	polycythaemia*:ab,ti	125
9	polycythemia*:ab,ti	polycythemia*:ab,ti	612
10	PV:ab,ti	PV:ab,ti	2581
11	#1 OR #2 OR #3 OR #4 OR #5 OR #6 OR #7 OR #8 OR #9 OR #10	([mh "Polycythemia Vera"] OR [mh "Polycythemia"] OR (polycythaemia next vera*):ab,ti OR (polycythemia next vera*):ab,ti OR (vaquez next disease*):ab,ti OR erythraemia*:ab,ti OR erythremia*:ab,ti OR polycythaemia*:ab,ti OR polycythemia*:ab,ti OR PV:ab,ti)	3092
12	"ropeginterferon alfa 2b":ab,ti	"ropeginterferon alfa 2b":ab,ti	58
13	ropeg*:ab,ti	ropeg*:ab,ti	74
14	#12 OR #13	("ropeginterferon alfa 2b":ab,ti OR ropeg*:ab,ti)	74
15	#11 AND #14	(([mh "Polycythemia Vera"] OR [mh "Polycythemia"] OR (polycythaemia next vera*):ab,ti OR (polycythemia next vera*):ab,ti OR (vaquez next disease*):ab,ti OR erythraemia*:ab,ti OR erythremia*:ab,ti OR polycythaemia*:ab,ti OR polycythemia*:ab,ti OR PV:ab,ti) AND ("ropeginterferon alfa 2b":ab,ti OR ropeg*:ab,ti))	50
16	MeSH descriptor: [Animals] explode all trees	[mh "Animals"]	903248
17	MeSH descriptor: [Humans] explode all trees	[mh "Humans"]	901941
18	#16 NOT #17	([mh "Animals"] NOT [mh "Humans"])	1307

19	#15 NOT #18	((([mh "Polycythemia Vera"] OR [mh "Polycythemia"] OR (polycythaemia next vera*):ab,ti OR (polycythemia next vera*):ab,ti OR (vaquez next disease*):ab,ti OR erythraemia*:ab,ti OR erythremia*:ab,ti OR polycythaemia*:ab,ti OR polycythemia*:ab,ti OR PV:ab,ti) AND ("ropeginterferon alfa 2b":ab,ti OR ropeg*:ab,ti)) NOT ([mh "Animals"] NOT [mh "Humans"]))	50
20	English:la	English:la	2181295
21	#19 AND #20	((([mh "Polycythemia Vera"] OR [mh "Polycythemia"] OR (polycythaemia next vera*):ab,ti OR (polycythemia next vera*):ab,ti OR (vaquez next disease*):ab,ti OR erythraemia*:ab,ti OR erythremia*:ab,ti OR polycythaemia*:ab,ti OR polycythemia*:ab,ti OR PV:ab,ti) AND ("ropeginterferon alfa 2b":ab,ti OR ropeg*:ab,ti)) NOT ([mh "Animals"] NOT [mh "Humans"])) AND English:la	50

Limit: English language; human studies.

Table S4. Risk-of-bias assessment of included studies (RoB 2 and ROBINS-I)

Study Type	Study	Randomization			Deviations from intended interventions	Missing outcome data	Measurement of outcome	Selection of reported result	Overall	Tool
Randomized Clinical Study	Gisslinger et al. (2020) (11)	Low			Some concerns	Some concerns	Low	Some concerns	Some concerns	RoB2
	Barbui et al. (2021) (12)	Low			Some concerns	Low	Low	Low	Low	RoB2
**Study Type**	**Study**	**Confounding**	**Selection of participants**	**Classification of interventions**	**Deviations from intended interventions**	**Missing data**	**Measurement of outcomes**	**Selection of reported result**	**Overall**	**Tool**
Non-Randomized Study	Yoon et al. (2025) (13)	Serious	Moderate	Low	Low	Moderate	Low	Moderate	Serious	ROBINS-I
	Palandri et al. (2024) (14)	Serious	Serious	Low	Moderate	Moderate	Low	Moderate	Serious	ROBINS-I
	SUO et al. (2024) (15)	Serious	Moderate	Low	Moderate	Moderate	Low	Moderate	Serious	ROBINS-I
	Chang et al. (2025) (16)	Serious	Serious	Moderate	Moderate	Moderate	Low	Moderate	Serious	ROBINS-I
	Edahiro et al. (2022) (17)	Serious	Moderate	Low	Low	Moderate	Low	Moderate	Serious	ROBINS-I
	Chen et al. (2024) (18)	Serious	Serious	Moderate	Moderate	Moderate	Low	Moderate	Serious	ROBINS-I
	Gisslinger et al. (2015) (19)	Serious	Moderate	Low	Moderate	Moderate	Low	Moderate	Serious	ROBINS-I

RoB 2, Risk of Bias 2 (Low risk of bias / Some concerns / High risk of bias); ROBINS-I, Risk Of Bias In Non-randomized Studies of Interventions (Low / Moderate / Serious / Critical / No information)

Table S5. Subgroup analyses comparing slow dose-up (SDU) and rapid dose-up (RDU) strategies for efficacy and safety outcomes

Outcomes	f/u time	Group	# of studies	Proportion				p-value for the comparison btw SDU and RDU
				estimate	Lower	Upper	p-value	
**CHR**	**1years**	Overall	9	0.59	0.47	0.71	<0.001	
		SDU	3	0.41	0.30	0.52	<0.001	0.000
		RDU	3	0.67	0.59	0.74	<0.001	
	**2years**	Overall	6	0.70	0.64	0.76	<0.001	
		SDU	2	0.63	0.54	0.72	<0.001	0.038
		RDU	3	0.75	0.68	0.82	<0.001	
	**3years**	Overall	3	0.73	0.65	0.81	<0.001	
		SDU	2	0.74	0.64	0.82	<0.001	
		RDU						
**MR**	**1years**	Overall	7	0.40	0.28	0.53	<0.001	
		SDU	3	0.36	0.29	0.43	<0.001	<0.001
		RDU	2	0.59	0.50	0.68	<0.001	
	**2yeaars**	Overall	6	0.69	0.54	0.83	<0.001	
		SDU	2	0.48	0.39	0.58	<0.001	0.292
		RDU	3	0.77	0.69	0.84	<0.001	
	**3years**	Overall	2	0.70	0.61	0.79	<0.001	
		SDU	1	0.68	0.58	0.77	<0.001	
**Absolute_JAK2_allele_ burden_Changes___1y**		Overall	5	-22.21	-34.06	-10.36	0.000	
		SDU	2	-14.264	-21.503	-7.024	0.000	0.007
		RDU	2	-35.105	-48.296	-21.915	<0.001	

**Thrombosis**	Overall	9	0.04	0.02	0.07	<0.001	
	SDU	3	0.04	0.02	0.08	<0.001	0.82
	RDU	3	0.04	0.02	0.09	<0.001	
**AE**	Overall	9	0.11	0.06	0.21	<0.001	
	SDU	3	0.18	0.06	0.41	0.011	0.56
	RDU	3	0.12	0.05	0.27	<0.001	
**SAE**	Overall	9	0.05	0.03	0.08	<0.001	
	SDU	3	0.05	0.02	0.13	<0.001	0.901
	RDU	3	0.05	0.03	0.10	<0.001	
**Discontinutions**	Overall	9	0.07	0.04	0.12	<0.001	
	SDU	3	0.11	0.05	0.23	<0.001	0.013
	RDU	3	0.02	0.01	0.06	<0.001	
**Transformation**	Overall	9	0.01	0.01	0.03	<0.001	
	SDU	3	0.01	0.00	0.04	<0.001	0.732
	RDU	3	0.01	0.00	0.05	<0.001	

Table S6. PRISMA Checklist

Section and Topic	Item #	Checklist item	Location where item is reported
**TITLE**			
Title	1	Identify the report as a systematic review.	Title page
**ABSTRACT**			
Abstract	2	See the PRISMA 2020 for Abstracts checklist.	Abstract
**INTRODUCTION**			
Rationale	3	Describe the rationale for the review in the context of existing knowledge.	Introduction
Objectives	4	Provide an explicit statement of the objective(s) or question(s) the review addresses.	Introduction
**METHODS**			
Eligibility criteria	5	Specify the inclusion and exclusion criteria for the review and how studies were grouped for the syntheses.	Methods – Eligibility Criteria
Information sources	6	Specify all databases, registers, websites, organisations, reference lists and other sources searched or consulted to identify studies. Specify the date when each source was last searched or consulted.	Methods – Systematic Review
Search strategy	7	Present the full search strategies for all databases, registers and websites, including any filters and limits used.	Supplementary Tables S1–S3
Selection process	8	Specify the methods used to decide whether a study met the inclusion criteria of the review, including how many reviewers screened each record and each report retrieved, whether they worked independently, and if applicable, details of automation tools used in the process.	Methods – Systematic Review
Data collection process	9	Specify the methods used to collect data from reports, including how many reviewers collected data from each report, whether they worked independently, any processes for obtaining or confirming data from study investigators, and if applicable, details of automation tools used in the process.	Methods – Data Extraction
Data items	10a	List and define all outcomes for which data were sought. Specify whether all results that were compatible with each outcome domain in each study were sought (e.g. for all measures, time points, analyses), and if not, the methods used to decide which results to collect.	Methods – Data Extraction; Table 1
	10b	List and define all other variables for which data were sought (e.g. participant and intervention characteristics, funding sources). Describe any assumptions made about any missing or unclear information.	Methods – Data Extraction; Table 1
Study risk of bias assessment	11	Specify the methods used to assess risk of bias in the included studies, including details of the tool(s) used, how many reviewers assessed each study and whether they worked independently, and if applicable, details of automation tools used in the process.	Methods – Systematic Review; Supplementary Table S4
Effect measures	12	Specify for each outcome the effect measure(s) (e.g. risk ratio, mean difference) used in the synthesis or presentation of results.	Methods- Meta-analysis

Section and Topic	Item #	Checklist item	Location where item is reported
Synthesis methods	13a	Describe the processes used to decide which studies were eligible for each synthesis (e.g. tabulating the study intervention characteristics and comparing against the planned groups for each synthesis (item #5)).	Studies were grouped by dosing strategy and follow-up time to determine eligibility for each synthesis.
	13b	Describe any methods required to prepare the data for presentation or synthesis, such as handling of missing summary statistics, or data conversions.	Data were extracted as reported; no imputation was performed. When necessary, outcomes were harmonized across studies based on commonly used definitions.
	13c	Describe any methods used to tabulate or visually display results of individual studies and syntheses.	Results were tabulated in summary tables and visualized using forest and line plots.
	13d	Describe any methods used to synthesize results and provide a rationale for the choice(s). If meta-analysis was performed, describe the model(s), method(s) to identify the presence and extent of statistical heterogeneity, and software package(s) used.	Random-effects meta-analysis (REML) was performed in R; heterogeneity assessed using I^2 and Q statistics.
	13e	Describe any methods used to explore possible causes of heterogeneity among study results (e.g. subgroup analysis, meta-regression).	Heterogeneity was explored using predefined subgroup analyses (SDU vs RDU).
	13f	Describe any sensitivity analyses conducted to assess robustness of the synthesized results.	No additional sensitivity analyses were conducted.
Reporting bias assessment	14	Describe any methods used to assess risk of bias due to missing results in a synthesis (arising from reporting biases).	Methods – Meta-analysis; Results – Publication bias (Begg's test)
Certainty assessment	15	Describe any methods used to assess certainty (or confidence) in the body of evidence for an outcome.	No formal assessment of certainty (e.g., GRADE) was conducted.
**RESULTS**			
Study selection	16a	Describe the results of the search and selection process, from the number of records identified in the search to the number of studies included in the review, ideally using a flow diagram.	Figure 1
	16b	Cite studies that might appear to meet the inclusion criteria, but which were excluded, and explain why they were excluded.	Figure 1
Study characteristics	17	Cite each included study and present its characteristics.	Table 1
Risk of bias in studies	18	Present assessments of risk of bias for each included study.	Supplementary Table-S4
Results of	19	For all outcomes, present, for each study: (a) summary statistics for each group (where appropriate) and	Figure 2-4

Section and Topic	Item #	Checklist item	Location where item is reported
individual studies		(b) an effect estimate and its precision (e.g. confidence/credible interval), ideally using structured tables or plots.	
Results of syntheses	20a	For each synthesis, briefly summarise the characteristics and risk of bias among contributing studies.	Supplementary Table-S4
	20b	Present results of all statistical syntheses conducted. If meta-analysis was done, present for each the summary estimate and its precision (e.g. confidence/credible interval) and measures of statistical heterogeneity. If comparing groups, describe the direction of the effect.	Random-effects meta-analyses reported with pooled estimates, 95% CI, heterogeneity (I^2), and subgroup comparisons (SDU vs RDU).
	20c	Present results of all investigations of possible causes of heterogeneity among study results.	Heterogeneity explored using subgroup analyses by dose-escalation strategy and follow-up duration; regional and design-related differences considered qualitatively.
	20d	Present results of all sensitivity analyses conducted to assess the robustness of the synthesized results.	No formal sensitivity analyses; extended follow-up data (2–3 years) synthesized separately to assess robustness.
Reporting biases	21	Present assessments of risk of bias due to missing results (arising from reporting biases) for each synthesis assessed.	Results – Begg's test
Certainty of evidence	22	Present assessments of certainty (or confidence) in the body of evidence for each outcome assessed.	We did not formally assess the certainty of evidence (e.g., using GRADE), as most included studies were non-randomized and substantial heterogeneity was present.
**DISCUSSION**			
Discussion	23a	Provide a general interpretation of the results in the context of other evidence.	Discussion
	23b	Discuss any limitations of the evidence included in the review.	Discussion
	23c	Discuss any limitations of the review processes used.	Discussion
	23d	Discuss implications of the results for practice, policy, and future research.	Discussion
**OTHER INFORMATION**			
Registration and protocol	24a	Provide registration information for the review, including register name and registration number, or state that the review was not registered.	This review was not registered.
	24b	Indicate where the review protocol can be accessed, or state that a protocol was not prepared.	A review protocol was not prepared.

Section and Topic	Item #	Checklist item	Location where item is reported
	24c	Describe and explain any amendments to information provided at registration or in the protocol.	No amendments were applicable.
Support	25	Describe sources of financial or non-financial support for the review, and the role of the funders or sponsors in the review.	Funding
Competing interests	26	Declare any competing interests of review authors.	Declarations – Competing interests
Availability of data, code and other materials	27	Report which of the following are publicly available and where they can be found: template data collection forms; data extracted from included studies; data used for all analyses; analytic code; any other materials used in the review.	Declarations – Availability of data and materials

From: Page MJ, McKenzie JE, Bossuyt PM, Boutron I, Hoffmann TC, Mulrow CD, et al. The PRISMA 2020 statement: an updated guideline for reporting systematic reviews. BMJ 2021;372:n71. doi: 10.1136/bmj.n71. This work is licensed under CC BY 4.0. To view a copy of this license, visit https://creativecommons.org/licenses/by/4.0/
